# Features in the Lipid Status of Two Generations of Fingerlings (0+) of Atlantic Salmon (*Salmo salar* L.) Inhabiting the Arenga River (Kola Peninsula)

**DOI:** 10.3390/ijms160817535

**Published:** 2015-07-30

**Authors:** Nina N. Nemova, Svetlana A. Murzina, Zinaida A. Nefedova, Alexey E. Veselov

**Affiliations:** 1 Environmental Biochemistry Lab, Institute of Biology, Karelian Research Centre of the Russian Academy of Sciences, Pushkinskaya st., 11, 185910 Petrozavodsk, Russia; E-Mails: nemova@krc.karelia.ru (N.N.N.); znefed@krc.karelia.ru (Z.A.N.); 2 Fish and Aquatic Invertebrate Ecology Lab, Institute of Biology, Karelian Research Centre of the Russian Academy of Sciences, Pushkinskaya st., 11, 185910 Petrozavodsk, Russia; E-Mail: veselov@krc.karelia.ru

**Keywords:** ontogenesis, generations, adaptations, lipids, fatty acids, *Salmo salar*, Kola Peninsula

## Abstract

The present research focused on determining the lipid status of salmon fingerlings (0+) in early development after dispersal form groups of spawning nests in biotopes of different hydrological conditions. The revealed qualitative and quantitative differences in the levels of phospholipids and fatty acids among two generations of Atlantic salmon fingerlings (0+) living in different biotopes of the Arenga River (a tributary of the Varzuga River) may be associated with the peculiarities of their genetically determined processes of the biosynthesis and modification of individual lipid classes and trophoecological factors (food spectrum, quality and availability of food objects, and hydrological regime). The research was organized to observe the dynamics of these developmental changes from ages 0+ to 2+.

## 1. Introduction

In the Russian North, the largest stock of Atlantic salmon (*Salmo salar* L.) reproduces in the Varzuga River in the basin of the White Sea (Kola Peninsula). From 25 to 70 thousand salmon spawn annually in the river. Such a large number of spawning migrants is associated not only with the large area but also with the quality of the spawning and nursery areas defined by the following main factors: slope, bottom configuration, fractional composition of the soil, depths and flow velocities [1,2]. In the Varzuga River, the spawning and nursery areas are located in the body as well as in numerous tributaries. In the year following spawning, when the spring flood is completed and water temperature increases up to 11–12 °C, salmon alevins that have hatched appear on the shingly bottom surface and begin to settle in their future places of habitation. This process is largely randomly determined, with active-passive dispersal in microsites characterized by different hydraulic and feeding conditions [3]. The habitat conditions of the juvenile salmon will then affect their survivability, growth rate and smoltification age.

Food and water temperature are also important factors. Temperature is the main abiotic determinant regulating the duration of the growth period [4] and the beginning of smoltification [5,6]. Knowledge of the ecological and biochemical mechanisms of the early development of Atlantic salmon (*Salmo salar* L.) is necessary to identify the features of the species’ development, the regularities of retarded or accelerated growth, and the beginning of the smoltification period. It is also of great interest for determining the role of the biotope’s environmental factors in producing the intrapopulation structure of the species in early development. Habitat conditions largely affect the physiological and biochemical states of aquatic organisms, including the level of lipids that have important functions in cellular metabolism.

In this context, the aim of this research was to determine the lipid status of salmon fingerlings (0+) in early development after dispersal form groups of spawning nests in biotopes with differing hydrological conditions. It was undertaken to observe the dynamics of such changes in development from ages 0+ to 2 + and further studies with bigger fish (3+ and smolts) will be done.

## 2. Results and Discussion

**Figure 1:**
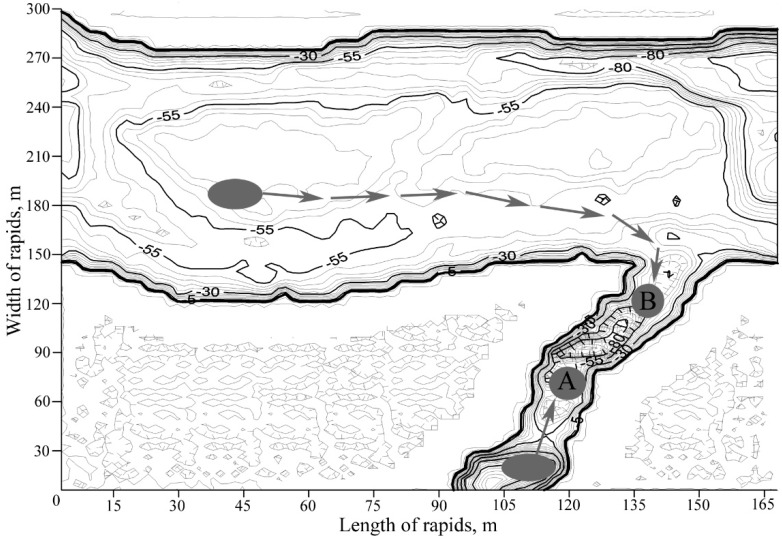
Hatching locations of the juvenile salmon in the Arenga tributary (marked as an oval from which the generation A formed) and mainstream of the Varzuga River (marked as an oval from which the generation B formed). Arrows show the migration of fingerlings and origins of the two generations.

We compared the lipid status of two generations of salmon fingerlings (0+) of equal size and weight (length 3.05 ± 0.02 and 3.09 ± 0.02 cm; weight 0.18 ± 0.01 and 0.19 ± 0.01 mg). Generation “A” was born in the Arenga River (a tributary of the Varzuga River) and slid to the riffle under the waterfall of this tributary. After hatching in the body of the Varzuga River, the juvenile fish of generation “B” moved to the mouth of the same Arenga tributary. Thus, two generations with different origins began to inhabit the same tributary (Figure 1).

However, the compared biotopes of their habitats had different hydrological, trophic and temperature conditions (Table 1) that affected the lipid status of the juvenile fish.

**Table 1:** Characteristics of the biotopes and food shot intensities of juvenile fish from the two generations.

Generation	Characteristics of Biotopes				Number of Food Shots in 15 min (*n* = 30 + 30 Specimens)
	Ground Fractions, * %	Depth, m (min-max)	Water Velocity, m/sec	Water Temperature, °С	
Generation “А”	BB–45; MB–25; SB–17; P–47; S–3	0.67 ± 0.03 (0.61–0.74)	0.94 ± 0.12 (0.81–1.27)	17.10 ± 0.20 (16.80–17.70)	28.51 ± 2.40 (25.0–33.0)
Generation “B”	BB–5; MB–5; SB–38; P–47; S–5	0.31 ± 0.08 (0.15–0.45)	0.52 ± 0.11 (0.35–0.75)	17.0 ± 0.32 (16.51–17.60)	16.21 ± 3.70 (9.0–23.0)

* Size of bottom clasts big boulders, BB (26–50 cm), medium-size boulders, MB (11–25 cm); small boulders, SB (6–10 cm), pebble, P (0.5–5 cm), sand, S (≤0.4 cm).

The lipid status of the two generations of fingerlings (0+) showed significant differences in the levels of total lipids (9.9% and 10.7% of the dry weight, respectively), including cholesterol (CHOL) (3.2% and 3.2%), triacylglycerols (TAGs) (1.7% and 2.0%) and the amount of monoenoic fatty acids (MUFA) (31.9% and 31.1%, respectively). High concentrations of structural lipids (the total PLs) were revealed among the fingerlings from under the waterfall (generation “A”), with higher concentration of energetic lipids and cholesterol esters (ECHOL) among the fish from the mouth of the Arenga tributary (generation “B”). Such high levels of ECHOL and the ECHOL/CHOL ratio (two-fold greater) among the juvenile fish of generation “B” compared with generation “A” may be associated with increased intake of CHOL or ECHOL with food. It was shown that the biomass of food and the intensity of feeding of juvenile salmon in the tributary was significantly higher than in the main channel [7]. CHOL esterification processes play an important role in metabolism, and enhancement of this process is a compensatory-adaptive response to excess intake. It is known [8,9] that in cells, a balanced system regulates the concentration of free CHOL, in which part is esterified and forms the cellular depot of CHOL and FAs in the form of ECHOL, which are more universal storage deposits than TAGs and have greater reserve function. It is also notable that we found a direct correlation of increasing concentration of ECHOL and linoleic acid (the latter discussed below) among the juvenile fish from the mouth of the Arenga tributary (generation “B”). We assume that this correlation may be associated with the increased activity of the lecithin-cholesterol acyltransferase enzyme (LCAT) and intake of linoleic 18:2(ω-6) acid with food.

The increased level of total phospholipids (PLs) changes the CHOL/PL ratio, which is the main factor controlling the microviscosity and fluidity of biomembranes and affecting their functional activity [10]. Due to the high level of PLs among the fingerlings from under the stream waterfall (generation “A”), the CHOL/PLs ratio was lower (0.6) with the same level of CHOL compared with that index (0.77) among the juvenile fish inhabiting the mouth of the Arenga tributary (generation “B”). Variations in the CHOL/PLs ratio are one of the ways of regulating the cellular biomembrane state in the process by which an organism adapts to specific environmental conditions. It is known that a decrease in the optimal limits of CHOL/PLs is accompanied by an increase of the functional activity of cellular receptors and the transport speed of ions, metabolites and water through membranes [10,11]. This biochemical process can be of particular importance in the case of motor overactivity in the juvenile fish of generation “A” under conditions of intensive turbulent flow in the riffle area (Table 2). The prevalence of the total PL level in the fingerling generation from under the waterfall (generation “A”) is connected with a higher proportion of individual classes of PLs: phosphatidylcholine (PC), phosphatidylethanolamine (PEA), phosphatidylserine (PS), and sphingomyelin (SFM) (Table 2).

Such differences in the concentration of individual PL classes among fish in different biotopes permit survival under different combinations of ecological factors. It is known that the adaptive role of individual PLs in ectothermic organisms is markedly diverse, both in the functions they regulate and in the mechanisms underlying their activity. A set of changes in the individual phospholipid classes allows them to effectively regulate the activity of membrane-bound enzymes, and one reason for changing their activity may be modification of the lipid environment that often has particular specificity resulting from various environmental changes [12,13,14]. Quantitative variations in structural phospholipids (mainly, PEA, PS and LysoPC) and their most unsaturated fatty acids support and provide the necessary membrane “fluidity” and affect the functional activity of membrane-bound enzyme systems of fish. The processes of adaptation to changing environmental conditions (abiotic factors such as temperature, salinity, pressure, depth and photoperiod as well as biotic factors such as the food base) are associated with this characteristic [11,13,15,16].

**Table 2:** Total lipid and lipid class content of the salmon fingerlings of two generations (% dry weight).

	Generation “А”	Generation “B”
Lipids	*n* = 29	*n* = 30
Total lipids	10.69 ± 0.39	9.94 ± 0.26 *
PL	5.30 ± 0.21	4.59 ± 0.21 *
PI	0.30 ± 0.02	0.29 ± 0.02
PS	0.38 ± 0.02	0.06 ± 0.01 *
PEA	1.24 ± 0.05	0.47 ± 0.04 *
PC	3.30 ± 0.12	2.29 ± 0.12 *
LysoPC	0.15 ± 0.02	1.26 ± 0.10 *
SFM	0.04 ± 0.01	0.01 ± 0.00
TAG	2.02 ± 0.16	1.69 ± 0.13 *
ECHOL	0.22 ± 0.03	0.46 ± 0.05 *
CHOL	3.15 ± 0.19	3.21 ± 0.22
CHOL/PL	0.63 ± 0.03	0.77 ± 0.07

Data means: M ± m; * indicates significant differences (*p* ≤ 0.05) between two generations; *n*—number of fish used in the analysis.

For various enzymes, certain PLs, which exhibit optimal activity in response to changes in environmental conditions, are required [12]. Thus, the increased content of minor phospholipids among the fingerlings from under the waterfall (generation “A”) with its intense flow may be associated with the regulatory influence of this PL on the activity of the *Na^+^*, *K^+^-ase* enzyme complex—the key enzyme of osmoregulation that is significant for a particular habitat [13]. The level of minor SFM is also increased among the fingerlings of generation “A”, which apparently indicates stabilization of the lipid bilayer due to high saturation of its FA composition. As a result, the oxidation of membrane lipids is reduced [17]. In addition to their structural function, individual classes of phospholipids (PS, SFM, LysoPC) participate as a second messenger in many cellular processes, including those under stressful environmental influences, which are accompanied by the strengthening of one or another physiological functions of the organism [10,18,19].

Lipids have important functions in all stages of ontogeny; their functions are largely determined by the spectrum of their fatty acid components, which most quickly become involved in the adaptive reactions of organisms [12,14,20].

In comparison with the generation “A” fingerlings from under the waterfall, the fingerlings of generation “B” from the mouth of the Arenga tributary exhibited higher levels of essential linoleic 18:2(ω-6) acid, which is typical of coastal insects included in the diet of juvenile salmon (Table 3).

**Table 3:** Fatty acids (FA) composition (% of total FAs) of the fingerlings of salmon from two generations.

	Generation “А”	Generation “B”
**FAs**	***n* = 26**	***n* = 30**
16:0	18.23 ± 0.78	18.87 ± 1.05
18:0	6.99 ± 0.42	7.89 ± 0.49
Sum MUFA	28.53 ± 0.73	30.54 ± 1.76 *
16:1(ω-7)	5.03 ± 1.00	5.16 ± 0.65
18:1(ω-9)	16.90 ± 1.85	16.93 ± 2.01
18:1(ω-7)	4.54 ± 0.36	4.73 ± 0.47
Sum MUFA	31.09 ± 1.86	31.93 ± 2.59
18:2(ω-6)	2.77 ± 0.72	3.63 ± 0.58 *
20:4(ω-6)	0.17 ± 0.07	0.21 ± 0.10
Sum (ω-6) PUFA	4.52 ± 1.02	5.30 ± 0.73
18:3(ω-3)	2.20 ± 0.67	2.19 ± 0.49
20:5(ω-3)	7.78 ± 0.53	7.33 ± 0.83
22:6(ω-3)	15.84 ± 1.81	13.34 ± 1.94 *
Sum (ω-3) PUFA	34.09 ± 2.16	30.54 ± 3.10 *
Sum PUFA	40.38 ± 1.79	37.53 ± 3.08 *

Data means: M ± m; * indicates significant differences (*p* ≤ 0.05) between the two generations, *n*—number of fish used in the analysis. FA discussed in the paper and some abundances are presented in the table. Sum of MUFA includes fatty acids: 10:0, 12:0, 14:0, 16:0, 18:0, 20:0, 24:0; Sum of MUFA includes: 14:1(n−5), 16:1(n−5), 16:1(n−9), 16:1(n−7), 17:1(n−7), 18:1(n−9), 18:1(n−7), 18:1(n−5), 20:1(n−11), 20:1(n−7), 20:1(n−9), 22:1(n−9), 22:1(n−11), 16:2(n−9), 18:2(n−9), 20:2(n−9), 22:2(n−9), 24:1(n−9); Sum of PUFA: 16:2(n−6), 20:2(n−6), 18:4(n−3), 20:4(n−3), 18:3(n−3), 20:5(n−3), 22:6(n−3), 16:3(n−6), 18:2(n−6), 18:3(n−6), 20:4(n−6).

The results reveal different metabolisms for polyunsaturated fatty acids (PUFA) among the fingerlings of the two generations. Their consumption by juvenile fish apparently resulted in a high 18:2(ω-6) acid content, which can be synthesized de novo in some species of insects [21]. It is in the tributary mouth under conditions of a water meadow where a massive development of flying insects takes place, which serve as food for the juvenile fish of generation “B”. No such conditions exist in the forest zone inhabited by the fish of generation “A”, and their food is mostly traditional—benthic invertebrates.

Among the fingerlings from under the waterfall of the Arenga tributary, we found high contents of polyunsaturated ω-3 fatty acids, mainly due to docosahexaenoic 22:6(ω-3) acid, and these high contents may be one of the biochemical adaptation mechanisms associated with the specific habitat conditions and hydrological regime (stream depth) (Table 1). The results indicate that among the juveniles of generation “A” from under the waterfall with more enhanced motor activity, the elongation and desaturation of food (ω-3) polyunsaturated fatty acids, mainly linoleic 18:3(ω-3) acid—a metabolic precursor of 22:6(ω-3) acid, the level of which was higher in this group—are accelerated. Some scientists relate increased levels of 22:6(ω-3) acid in the lipids of fish muscles with enhanced motor activity [12,22,23,24]. For example, high concentrations of 22:6(ω-3) acid were observed among tunas (*Thunnus* ssp.) and flying fish (*Exocoetidae family*) (up to 20.8% of the total FA), in the muscles of juvenile daubed shanny (*Leptoclinus maculatus*) [25] and in muscles of the Atlantic bonito (*Sarda sarda*) [26] that undergo intensive motor activity. In less mobile forms (conger eels (*Congridae* family), scorpion fish (*Scorpaenidae* family), halibut (*Hippoglossus hippoglossus*)), the proportion of this acid does not exceed 5.8%–7.8% [22]. It is not coincidental that docosahexaenoic 22:6(ω-3) acid is called the “mechanism of adaptation” in fish [17] and is the most genetically determined lipid [27]. In cold-blooded organisms, compensatory processes are primarily connected with changing the fatty acid composition of biomembrane lipids.

Elevated levels of (ω-3) PUFA, due mainly to 22:6(ω-3) acid, among the juvenile fish from under the waterfall (generation “A”) compared with the fish from the mouth of the Arenga tributary (generation “B”) are correlated with a higher proportion of phospholipids such as PEA, PC and PS, which decrease the CHOL/PLs ratio and could contribute to enhanced functional activity of cell receptors and indirectly increase the transport speed of ions, metabolites and water. According to some authors [11,16], in juvenile salmon in differing habitat conditions, variations in the level of individual classes of PLs and their FAs may indicate changes in the “fluidity” of biomembranes and the specific regulation of related enzymes in accordance with changing environmental conditions.

## 3. Experimental Section

Salmon fingerlings (0+) were caught in the nursery areas of microbiotopes in the Arenga River (a tributary of the Varzuga River) during the period when they had formed stable groups. An electrofishing device (Fa-2, Norway) was used for this purpose. Every sample included from 13 to 30 fingerlings. Before sampling, underwater observations of the intensity of feeding of the juvenile fish of both groups were carried out. The number of food shots within 15 min was counted for every juvenile fish, and after sampling, the characteristics of the biotopes were recorded (depth, flow velocity, type of substrate) (Table 1). To avoid the effect of electrofishing, fingerlings were held for 24 h in cages located in the mainstream portion of the river.

The fish were homogenized in 10 volumes (10 mL each) of 96% ethyl alcohol mixed with 0.001% of the antioxidant (ionol). The homogenates were placed in glass vials and delivered to the laboratory. The material was then fixed in a solvent of chloroform: methanol (2:1, *v*/*v*), and the total lipids (TLs) were extracted using the method previously described [28]. The sample under analysis was filtered, and the residue retained on the paper filter was rinsed with extractive mixture at room temperature; to remove water-soluble impurities. The chloroform-methanol mixture and the pure chloroform were added. Finally, the distilled water was added in the glass flat-bottomed flask with the sample lipid extract. The flask with the sample lipid extract was vortex and stirred, and left to settle in the glass separating funnel until complete separation of phases.

Lipids remain in the lower chloroform layer, whereas non-lipid substances move to the upper aqueous methanol phase. The chloroform layer was then withdrawn to be evaporated under vacuum on a rotary evaporator. The residues recovered after lipid extraction from the tissues were dried over phosphoric anhydride until the samples reached a constant weight. The residues were weighed (X1) to determine the approximate percentage of total lipid on a dry-weight basis:

Total lipids (% dry weight) = X2 × 100/(X1 + X2), where X1 = residue weight (g) and X2 = lipid extracted (g).

The lipid status of each fish was evaluated by determining the content of total lipids, triacylglycerides (TAGs), and phospholipids (PLs), including the separate phospholipid classes phosphatidylcholine (PC), phosphatidylethanolamine (PEA), phosphatidylserine (PS), phosphatidylinositol (PI), lysophosphatidylcholine (LysoPC) and sphingomyelin (SFM), cholesterol (CHOL), cholesterol esters (ECHOL) and the fatty acid (FA) spectrum.

Thin-layer chromatography was used to identify the lipid classes: PLs, TAGs, CHOL and ECHOL; 15 µL of the total lipids extract from each sample was loaded on the TLC plate. After drying, the chromatogram was developed in iodine vapor, which stains lipids yellow. These molecules were quantified using a modified hydroxamate method [29], which involves the formation of dark-brown complexes of trivalent iron ions with hydroxamic acid through ester bonding between the lipids and hydroxylamine [30]. The stain intensity was measured using a spectrophotometer (Spektr, St. Petersburg, Russia, SF-2000) at a wavelength of 540 nm. The quantitative determination of CHOL was determined according to the method described in [31] using trichloroacetic iron dissolved in perchloric acid. The stain intensity was measured using a spectrophotometer at a wavelength of 550 nm. Standards (Sigma Aldrich, St. Louis, MO, USA) for thin-layer chromatography were used to distinguish the lipid classes on the plates.

The chromatograms of individual phospholipid fractions were determined by high-performance liquid chromatography (HPLC) according to the method of [32] using a Nucleosil 100-7 column (Macherey-Nagel GmbH & Co., Duren, Germany) with a acetonitrile:hexane:methanol:phosphorus acid (918:30:30:17.5 by volume) mobile phase. The detection was performed using a spectrophotometer (UV light, 206 nm). Phospholipid standards (Supelco, Bellefonte, PA, USA) were used for the identification and quantification of the phospholipid compounds in the sample. We identified six phospholipids: phosphatidylserine, phosphatidylethanolamine, phosphatidylinositol, phosphatidylcholine, lysophosphatidylcholine and sphingomyelin.

The fatty acid composition of the total lipid extracts was analyzed by gas-liquid chromatography. Fatty acid methyl esters (FAMEs) were identified using a “Chromatek-Crystall-5000.1” (Chromatek, Yoshkar-Ola, Russia) gas chromatograph with a flame-ionization detector and a Zebron capillary gas chromatographic column (Phenomenex, Torrance, CA, USA). An isothermal column configuration was used (205 °C); the temperatures of the detector and evaporator were 250 and 240 °C, respectively. The internal standard was 22:0 FA. Chromatek-Analytik-5000.2 software (Chromatek, Yoshkar-Ola, Russia) was used for recording and integrating the data. FAMEs were identified with standard mixtures Supelco 37 FAME mix (Supelko, Bellefonte, PA, USA) and by comparing the equivalent lengths of carbon chains and table constants according to Jamieson [33].

The research was carried out using the facilities of the Equipment Sharing Centre of the Institute of Biology, KarRC of RAS. The results are given as the M ± m (average ± SD). The data were analyzed to determine whether they exhibited a normal distribution. The differences between the means of the lipid and fatty acid parameters of the fish rom two generations were tested by ANOVA (*p* ≤ 0.05). Tukey’s honestly significance test (HSD) was applied for comparisons. The StatGraf 2.5 statistic package was used for graphical presentation.

## 4. Conclusions

The qualitative and quantitative differences in lipid and fatty acid spectra among two generations of Atlantic salmon fingerlings (0+) living in different biotopes in the Arenga tributary were revealed. The levels of total lipids, triacylglycerols, certain phospholipids and fatty acids (sum of monoenic and docosahexaenoic fatty acid) were significantly higher in the juvenile fish of generation “A” in comparison to fish of generation “B” associated with trophoecological factors (food spectrum, quality and availability of food objects and hydrological regime) and indicated more favorable conditions in the tributary in general. At the same time, fish from two studied locations showed distinguishable features between biotopes and conditions for growth and development. Observed differences in the levels of cholesterol and cholesterol esters and certain fatty acids involved in their metabolism allow us to determine the specifics of their genetically determined processes of biosynthesis and effect of dietary intake. The data obtained permit us to suggest that the stability of the regulation of vital functions in different ecological conditions is provided by structural reconstructions of the lipid systems in organisms, which are due to changing ratios of individual lipid classes and fatty acid radicals.

In the future, it will be interesting to see whether such lipid status heterogeneity persists and what the dynamics of the lipid parameters may be in the further development of juvenile fish from ages 0+ to 2+ and then to 3+ and smolts in future research planned. Study of the biochemical aspects of early development will allow a thorough understanding and assessment of the adaptive capacity of juvenile salmon.
